# Overlap Syndrome of Immune Reconstitution Inflammatory Syndrome Secondary to Disseminated Mycobacterial Infection and Hemophagocytic Lymphohistiocytosis in a Patient With Newly Diagnosed HIV/AIDS

**DOI:** 10.7759/cureus.53410

**Published:** 2024-02-01

**Authors:** Nishant Patel, Cody Chastain

**Affiliations:** 1 Infectious Diseases, Vanderbilt University Medical Center, Nashville, USA

**Keywords:** antiretroviral therapy, acquired immune deficiency syndrome, human immunodeficiency virus, iris, hlh, mycobacteria, aids, hiv

## Abstract

Immune reconstitution inflammatory syndrome (IRIS) following initiation of antiretroviral therapy (ART) has variable incidence but is not uncommon and has the potential to cause long-term consequences and fatal outcomes in patients with HIV. Hemophagocytic lymphohistiocytosis (HLH) is a separate syndrome of excess immune activation, but may coexist with IRIS and necessitate a unique treatment approach. In this report, the case of a patient with newly diagnosed HIV/AIDS who was found to have both mycobacterial IRIS and HLH is presented.

## Introduction

While the incidence of mycobacterium avium complex (MAC) disease has declined greatly with modern antiretroviral therapy (ART), patients with HIV who are not on effective ART or chemoprophylaxis remain a high-risk population for disease acquisition. The signs and symptoms of immune reconstitution inflammatory syndrome (IRIS) are often difficult to distinguish from active MAC infection. Additionally, the excess immune activation seen in hemophagocytic lymphohistiocytosis (HLH) can present with many of the same signs and symptoms seen in disseminated MAC infection or IRIS. Therefore, given that mycobacterial IRIS in patients with HIV has overlapping clinical features and pathogenesis with HLH, diagnosis and treatment can prove challenging.

## Case presentation

A 31-year-old male with a history of newly diagnosed HIV/AIDS and disseminated histoplasmosis was admitted for worsening abdominal pain. The pain began one week prior to admission and was described as a stabbing pain, worst in the left upper quadrant. He also endorsed subjective fevers, chills, diaphoresis, nausea, constipation, and nonproductive cough. He was admitted one month prior with similar symptoms and was found to have a new diagnosis of HIV/AIDS with a CD4 count of 54 cells/mm^3^ and a viral load of 952,000 copies/ml. CT abdomen/pelvis at that time demonstrated numerous bulky mesenteric and retroperitoneal lymph nodes, and a workup for opportunistic infections revealed a positive urine histoplasma antigen of 4.84 ng/ml. He was recently started on antiretroviral therapy (ART) with bictegravir/emtricitabine/tenofovir alafenamide (Biktarvy) after completing two weeks of itraconazole, along with trimethoprim-sulfamethoxazole for pneumocystis prophylaxis. He reported compliance with his medications and was feeling well until the recurrence of his symptoms one week prior to his current admission.

The patient was born in Mexico but had lived in Tennessee for over two years at the time of presentation. He worked in a factory. He denied a history of tobacco use, recreational drug use, or injection drug use. He drank about one alcoholic drink per week. He had no history of tattoos, prior surgeries, or blood transfusions. He had about 10 sexual partners till the time of presentation, both male and female. He reported two sexual partners in the past year.

The patient’s vitals on admission were as follows: temperature of 97.9°F (36.6°C), pulse of 107 beats per minute, blood pressure of 100/69 mmHg, respiratory rate of 16 breaths per minute, and oxygen saturation of 100% on room air. He was thin-appearing, diaphoretic, and in mild distress secondary to pain. Eye exam revealed mild conjunctival injection bilaterally. Head and neck exam revealed moist mucous membranes, a clear oropharynx, and no cervical adenopathy. Cardiovascular exam revealed tachycardia with regular rhythm and no murmurs, rubs, or gallops. Respiratory exam was clear to auscultation bilaterally. Abdominal exam revealed a soft, nondistended abdomen with mild tenderness to palpation in the left upper quadrant. Skin examination was without rashes or lesions. Neurological exam was nonfocal, with cranial nerves II-XII grossly intact. Psychiatric exam was notable for anxious mood. The remainder of the physical exam was otherwise normal.

Complete blood count was notable for white blood cells 4.7 x 10^3^/mcL (reference range 3.9-10.7x10^3^/mcL. Hemoglobin was 12 gm/dL (reference range 14.0-18.1 gm/dL). The platelet count was 231x10^3^/mcL (reference range 135-371x10^3^/mcL). Comprehensive metabolic panel was notable for a sodium level 133 mmol/L (reference range 136-145 mmol/L), potassium level 4.2 mmol/L (reference range 3.3-4.8mmol/L), chloride level 104 mmol/L (reference range 98-107 mmol/L), carbon dioxide level 24 mmol/L (reference range 22-29 mmol/L), glucose level 105 mg/dL (reference range 70-99 mmol/L). The creatinine level was 0.99 mg/dL (reference range 0.72-1.25 mg/dL). Liver function tests were notable for aspartate aminotransferase 42 unit/L (reference range 5-40 unit/L), alanine aminotransferase 37 unit/L (reference range 0-55 unit/L), alkaline phosphatase 158 unit/L (reference range 40-150 unit/L), and total bilirubin 0.4 mg/dL (reference range 0.2-1.2 mg/dL).

CT chest with contrast showed no focal consolidations, ground-glass opacities, or nodules. CT abdomen/pelvis with contrast showed the development of mild splenomegaly (new from prior CT one month prior) and a slight increase in moderate central mesenteric adenopathy with stable mild retroperitoneal adenopathy.

Given the recurrence of symptoms following the initiation of ART, there was concern for IRIS. However, the new splenomegaly and persistent abdominal lymphadenopathy warranted further investigation. Blood cultures, including fungal and acid-fast bacillus (AFB), remained negative. Workup for tuberculosis was negative, including AFB sputum cultures. He was started on liposomal amphotericin B in place of itraconazole due to concern that histoplasmosis may have been inadequately treated or be a contributor to possible IRIS. PET-CT demonstrated markedly F-18 fluorodeoxyglucose (FDG)-avid splenomegaly, right cervical lymphadenopathy, and abdominal adenopathy concerning for lymphoproliferative neoplasm. He subsequently underwent an excisional right cervical lymph node biopsy; pathology demonstrated atypical lymphoid tissue and inflammatory cells suggestive of a reactive process, likely related to underlying HIV, there was no evidence of malignancy, and AFB/fungal staining was negative.

**Figure 1:**
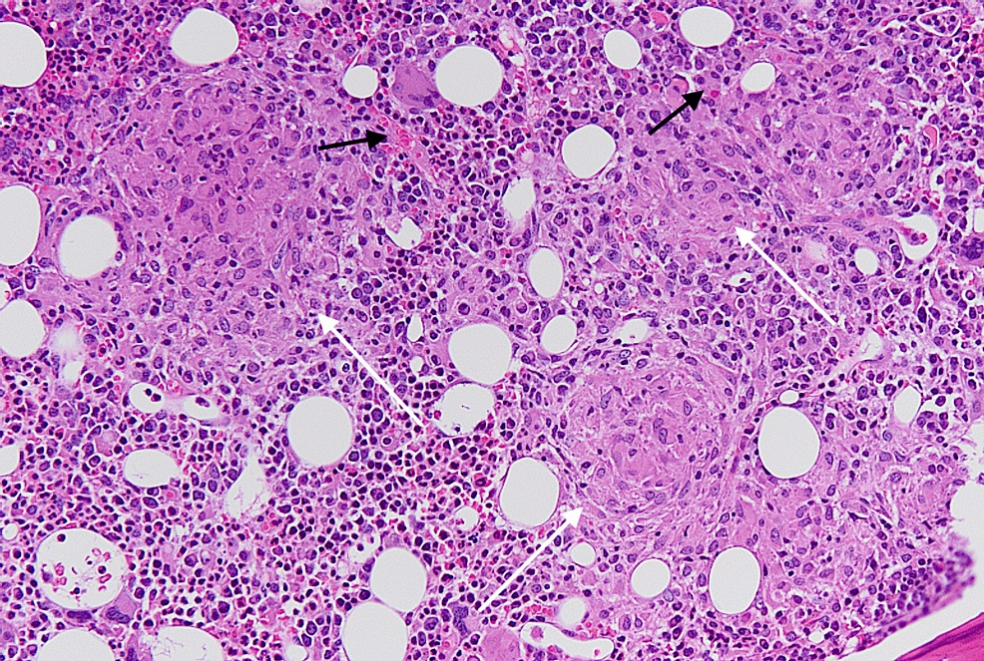
Bone marrow biopsy (H&E stain, 20x) showing hypercellular marrow with much of the marrow space occupied by non-necrotizing granulomas (white arrows). Occasional hemophagocytosis is present in the background (black arrows). H&E: Hematoxylin and eosin

**Figure 2:**
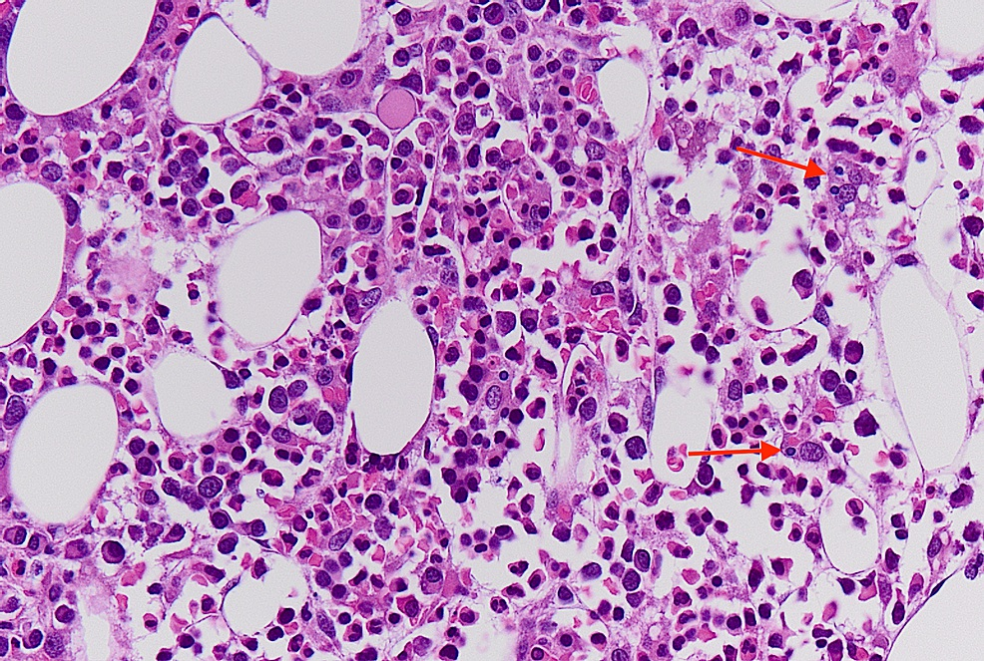
Bone marrow biopsy (H&E stain, 40x) showing hemophagocytosis in the marrow space (red arrows). H&E: Hematoxylin and eosin

**Figure 3:**
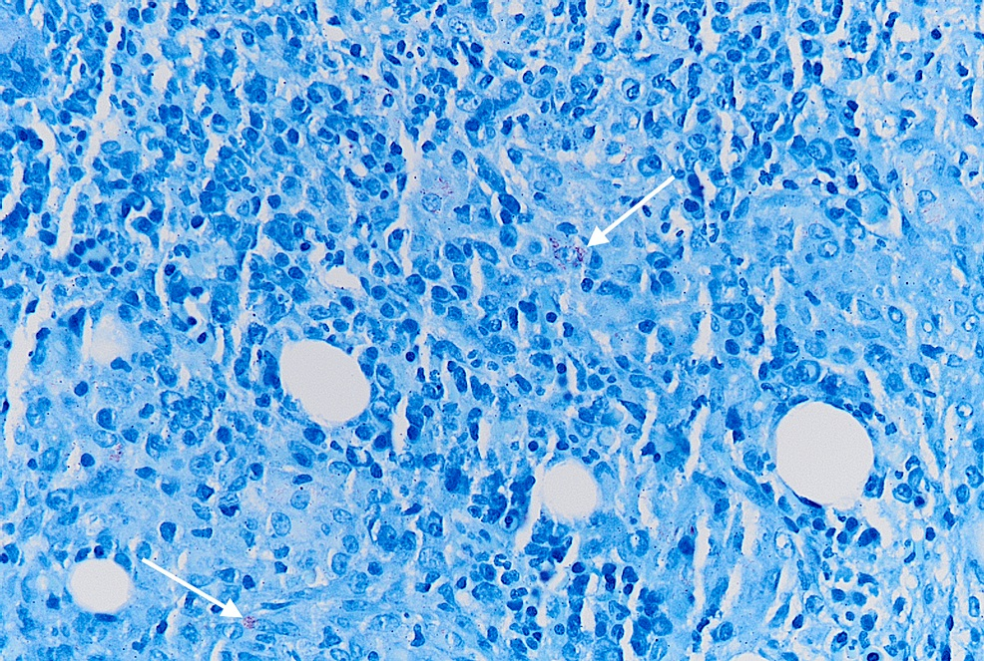
Bone marrow biopsy (AFB stain, 40x) showing non-necrotizing granulomas containing AFB-positive mycobacterial organisms (white arrows). AFB: Acid-fast bacilli

His hospital course was complicated by persistent fevers, worsening pancytopenia, acute kidney injury, cholestatic liver injury, and elevated ferritin of 2,256 ng/ml (reference range 24-336 ng/ml). Bone marrow biopsy was performed which revealed hypercellular marrow, with non-necrotizing granulomas filled with AFB-positive mycobacterial organisms occupying most of the marrow space, as well as occasional hemophagocytosis (Figures 1-3).

Liver biopsy revealed multiple portal tracts with epithelioid granulomas, one with focal caseating necrosis. Soluble interleukin-2 receptor (sIL-2R) level (a diagnostic marker in HLH) returned elevated at 13,910 U/mL (ref range 137-838 U/mL).

The biopsy results confirmed the diagnosis of IRIS, likely from unmasking of disseminated mycobacterial infection with likely secondary HLH. Cultures from bone marrow and biopsies remained negative, but MAC infection was thought to be most likely given his clinical picture.

For disseminated mycobacterial infection, he was started on azithromycin, ethambutol, moxifloxacin, and rifampin. For presumed secondary HLH and IRIS, he was started on a dexamethasone taper. Rifampin was eventually discontinued due to ongoing cholestatic liver injury. He was transitioned from liposomal amphotericin B to isavuconazole (due to a lower risk of hepatotoxicity than itraconazole) for treatment of disseminated histoplasmosis.

He was eventually discharged from the hospital on a dexamethasone taper to be completed over a few weeks, along with treatment for MAC, histoplasmosis, and HIV. He did well initially and completed his steroid taper with the resolution of his leukopenia and thrombocytopenia. However, about one to two weeks following the completion of steroids, he was re-admitted with syncopal episodes and recurrent fevers. Labs demonstrated WBC 0.2 x10^3^/mcL, hemoglobin 6.5 gm/dL, platelets 37 x10^3^/mcL, and ferritin 59,475 ng/mL (previously ~3000 ng/mL). Additional workup for new infections remained negative. His clinical presentation and laboratory findings were felt to be due to an overlap syndrome of IRIS and HLH, with acute worsening of HLH in the setting of recent steroid discontinuation. Therefore, he was re-initiated on a more aggressive steroid regimen, with a plan for a prolonged taper over several months.

While he initially tolerated the resumption of steroids, he had frequent hospital readmissions for neutropenic fever and HLH flares, along with significant side effects related to prolonged steroid use. Given steroid intolerance and limited treatment options in the setting of profound myelosuppression, anakinra was initiated. He tolerated this therapy with gradual improvement of fevers and cytopenias. 

## Discussion

MAC disease generally occurs in people with HIV with CD4 cell counts <50 cells/mm^3^. While the incidence of MAC disease continues to decline with modern ART, the incidence of disseminated disease is 20-40% in those not on effective ART or chemoprophylaxis [1]. In addition to low CD4 cell count, other risk factors associated with MAC disease include HIV RNA levels >1000 copies/mL, ongoing viral replication despite ART, or concurrent opportunistic infections (e.g. histoplasmosis) [2]. Symptoms of MAC disease can be nonspecific but generally include fever, night sweats, weight loss, weakness, diarrhea, or abdominal pain. Laboratory abnormalities associated with disseminated disease include anemia and elevated alkaline phosphatase levels [3]. Radiographic findings may include hepatosplenomegaly and lymphadenopathy (usually retroperitoneal, paratracheal, or para-aortic). Diagnosis of disseminated MAC disease is confirmed in those with compatible signs and symptoms along with isolation of MAC from cultures of normal sterile tissue or body fluids (e.g. blood, bone marrow, or lymph node). Preferred treatment includes at least two drugs (generally clarithromycin or azithromycin plus ethambutol) to prevent the emergence of resistance. In those with high mycobacterial loads or in those not on effective ART, a third or fourth drug may be added (generally a rifamycin, fluoroquinolone, or aminoglycoside) [4].

IRIS is a term used to describe a group of inflammatory disorders associated with paradoxical worsening of a preexisting infectious process following initiation of ART in HIV patients. The signs and symptoms of IRIS may be clinically indistinguishable from active MAC infection. IRIS may be “paradoxical” in those with known MAC disease or “unmasking” in those with undiagnosed MAC infection [5]. The syndrome usually occurs within a few weeks to a few months following initiation of ART in those with advanced HIV/immunosuppression who have a rapid and dramatic reduction in plasma HIV RNA level [6]. The clinical course may be self-limited or result in severe, unremitting symptoms requiring the use of systemic corticosteroids or other anti-inflammatory therapies.

HLH is another syndrome of excessive immune activation. While it is mostly seen in infants due to genetic defects, it can also be seen in adults, usually from an immunologic trigger such as infection, malignancy, or rheumatologic condition [7]. Patients with HLH present with many of the same signs and symptoms seen in disseminated MAC infection or IRIS, such as fever, hepatosplenomegaly, lymphadenopathy, and cytopenias. Patients also typically have markedly elevated serum ferritin levels [8]. The diagnosis is made in those with compatible symptoms in the setting of elevated inflammatory markers (e.g. ferritin, sIL-2R). There are formal diagnostic criteria published in the HLH-2004 trial [9].

Given that mycobacterial IRIS in patients with HIV has overlapping clinical features and pathogenesis with HLH, diagnosis and treatment can prove challenging. Rocco et al. recently studied a cohort of 80 patients with advanced HIV and mycobacterial infections; they found that up to one-third of patients with severe mycobacterial IRIS met the criteria for diagnosis of HLH [10]. Furthermore, this subgroup of patients with a dual diagnosis of IRIS and HLH had a unique and more severe clinical course requiring more steroids and a longer duration of immunosuppression, with many requiring additional therapies beyond steroids due to refractory symptoms or steroid intolerance. Therefore, identifying relevant clinical markers (e.g. hemoglobin, ferritin, sIL-2R, CXCL9) early on may improve risk stratification and lead to prompt initiation of steroids or other potential therapeutic targets.

## Conclusions

While IRIS secondary to mycobacteria or other opportunistic infections and HLH are distinct entities, they have overlapping pathogenesis and clinical presentation. Furthermore, many patients with mycobacterial IRIS also meet the criteria for diagnosis of HLH, and this population of patients has a more prolonged and severe clinical course refractory to standard treatments. Therefore, early identification of relevant clinical biomarkers may aid in isolating high-risk patient groups that could benefit from prompt initiation of steroids or other therapeutic targets. 
